# Risk factors for preeclampsia and eclampsia at a main referral maternity hospital in Freetown, Sierra Leone: a case-control study

**DOI:** 10.1186/s12884-021-03874-7

**Published:** 2021-06-02

**Authors:** N. Stitterich, J. Shepherd, M. M. Koroma, S. Theuring

**Affiliations:** 1 Institute of Tropical Medicine and International Health, Charité-Universitätsmedizin Berlin, corporate member of Freie Universität Berlin, Humboldt-Universität zu Berlin, and Berlin Institute of Health, Augustenburger Platz 1, 13353 Berlin, Germany; 2 National School of Midwifery, Freetown, Sierra Leone; 3 Princess Christian Maternity Hospital, Freetown, Sierra Leone

**Keywords:** Preeclampsia, Eclampsia, Risk factors, Freetown, Sierra Leone

## Abstract

**Background:**

In the African region, 5.6% of pregnancies are estimated to be complicated by preeclampsia and 2.9% by eclampsia, with almost one in ten maternal deaths being associated with hypertensive disorders. In Sierra Leone, representing one of the countries with the highest maternal mortality rates in the world, 16% of maternal deaths were caused by pregnancy-induced hypertension in 2016. In the light of the high burden of preeclampsia and eclampsia (PrE/E) in Sierra Leone, we aimed at assessing population-based risk factors for PrE/E to offer improved management for women at risk.

**Methods:**

A facility-based, unmatched observational case-control study was conducted in Princess Christian Maternity Hospital (PCMH). PCMH is situated in Freetown and is the only health care facility providing ‘Comprehensive Emergency Obstetric and Neonatal Care services’ throughout the entire country. Cases were defined as pregnant or postpartum women diagnosed with PrE/E, and controls as normotensive postpartum women. Data collection was performed with a questionnaire assessing a wide spectrum of factors influencing pregnant women’s health. Statistical analysis was performed by estimating a binary logistic regression model.

**Results:**

We analyzed data of 672 women, 214 cases and 458 controls. The analysis yielded several independent predictors for PrE/E, including family predisposition for PrE/E (AOR = 2.72, 95% CI: 1.46–5.07), preexisting hypertension (AOR = 3.64, 95% CI: 1.32–10.06), a high mid-upper arm circumflex (AOR = 3.09, 95% CI: 1.83–5.22), presence of urinary tract infection during pregnancy (AOR = 2.02, 95% CI: 1.28–3.19), presence of prolonged diarrhoea during pregnancy (AOR = 2.81, 95% CI: 1.63–4.86), low maternal assets (AOR = 2.56, 95% CI: 1.63–4.02), inadequate fruit intake (AOR = 2.58, 95% CI: 1.64–4.06), well or borehole water as the main source of drinking water (AOR = 2.05, 95% CI: 1.31–3.23) and living close to a waste deposit (AOR = 1.94, 95% CI: 1.15–3.25).

**Conclusion:**

Our findings suggest that systematic assessment of identified PrE/E risk factors, including a family predisposition for PrE/E, preexisting hypertension, or obesity, should be performed early on in ANC, followed by continued close monitoring of first signs and symptoms of PrE/E. Additionally, counseling on nutrition, exercise, and water safety is needed throughout pregnancy as well as education on improved hygiene behavior. Further research on sources of environmental pollution in Freetown is urgently required.

**Supplementary Information:**

The online version contains supplementary material available at 10.1186/s12884-021-03874-7.

## Background

Preeclampsia is defined as pregnancy-induced hypertension, occurring after 20 weeks of gestation, accompanied by new-onset proteinuria, maternal organ- or uteroplacental dysfunction [1]. Maternal organ dysfunction can manifest in symptoms like epigastric pain, visual disturbance, or severe headache. Uteroplacental dysfunction can lead to fetal growth restriction with low birth weight infants [1]. The complication of a generalized seizure in a preeclamptic woman is referred to as eclampsia and can lead to maternal death [2]. In the African region, 5.6% of pregnancies are estimated to be complicated by preeclampsia and 2.9% by eclampsia and hypertensive disorders in pregnant women are responsible for almost one in ten maternal deaths [3, 4]. While the etiology of preeclampsia and eclampsia (PrE/E) is still not fully understood, knowledge of specific risk factors for PrE/E in a selected healthcare setting is essential to identify women at risk. Established risk factors for PrE/E include nulliparity, multifetal gestation, previous abort/stillbirth, a history of PrE/E as well as a family predisposition of PrE/E and co-morbidities like chronic hypertension, pregestational diabetes, obesity, and urinary tract infections (UTIs) [5–10]. Considering large differences in living conditions, risk factors for PrE/E referred to by low- and middle-income countries as from the sub-Saharan African region can partly diverge from those from high-income countries. In sub-Saharan African countries, low educational levels were described as risk factors for PrE/E, and in a secondary analysis of a multi-country survey conducted by the World Health Organization (WHO), adolescent pregnancies significantly raised the risk of PrE/E [11–13]. In the Democratic Republic of Congo (DRC), Nigeria and Ghana, environmental pollution with heavy metals as well as psychosocial factors like emotional stress, poor sleeping quality and the intake of traditional treatment during pregnancy increased the risk of PrE/E [13–18]. In malaria-endemic countries like Central Sudan and Senegal, *Plasmodium falciparum*-infected placenta was described to influence the risk of PrE/E [19–21]. In Zimbabwe and Ethiopia, obesity measured with a high mid-upper arm circumflex (MUAC) heightened the risk for preeclampsia, while a regular intake of fruits and vegetables reduced the risk [22–26]. Sierra Leone is a resource-restricted West-African country with an estimated population of seven million, with Freetown as its capital [27]. In 2017, Sierra Leone reported one of the highest maternal mortality ratios in the world with 1120 maternal deaths in 100,000 live births [28]. In 2016, pregnancy-induced hypertension caused 16% of maternal deaths in Sierra Leone [29]. A recent multicenter study reporting from 10 low and middle-income countries described the incidence of eclampsia in Freetown as the highest of all study sites with 142 cases per 10,000 births and a case fatality rate of 15.5 per 10,000 births [30]. Although the burden of PrE/E in Sierra Leone is high, annual nationwide documentation and population-based risk factors for PrE/E are not available yet. Lacking risk factor assessment makes it impossible to target specific at-risk groups and improve their health outcomes by early monitoring and case management. Therefore, our study aimed to explore the potential risk factors for PrE/E within a cohort from a tertiary care facility located in Freetown, Sierra Leone.

## Methods

### Design and setting

We conducted a facility-based, unmatched observational case-control study among pregnant and postpartum women attending inpatient care or skilled delivery at Princess Christian Maternity Hospital (PCMH). PCMH is the main referral maternity hospital for the entire country as well as a teaching hospital for the University of Sierra Leone, situated in Freetown. PCMH is the only health care facility providing ‘Comprehensive Emergency Obstetric and Neonatal Care services’ throughout the Western Area representing 21% of the entire country population [31]. In 2018, 18.8% of major direct obstetric emergencies in PCMH and 14.7% of all maternal deaths were related to PrE/E [31]. Outcome indicators of our study included primarily population-based risk factors for PrE/E and secondarily clinical presentation and birth outcomes associated with PrE/E.

### Recruitment and study procedures

Between November 2018 and February 2019, pregnant and postpartum clients of PCMH were recruited according to the following eligibility criteria: A ‘case’ was defined as a pregnant or postpartum woman diagnosed with preeclampsia or eclampsia following National Protocols and Guidelines for Emergency Obstetric and Newborn Care published by the Ministry of Health of the Government of Sierra Leone in 2018 [32]. Those specify preeclampsia as a new onset of blood pressure ≥ 140/ 90 mmHg during at least two readings measured four hours apart at a time > 20 weeks of gestation with adding any of the following signs or symptoms: proteinuria of at least 2+ in urinalysis, headache, visual disturbance or generalized edema on hands or face. Eclampsia is defined as a complication of preeclampsia marked by generalized seizures and can be diagnosed without the elevated blood pressure and proteinuria if other conditions were ruled out in differential diagnoses, like epilepsy, cerebral malaria, meningitis, or hypoglycemia [32]. A ‘control’ was defined as a normotensive postpartum woman, who was not diagnosed with PrE/E at any time in this pregnancy. All study participants gave written consent and received detailed information about PrE/E as well as financial compensation. In agreement with the hospital administration of PCMH and the Ethical Committee of the Ministry of Health and Sanitation, Sierra Leone and Charité- Universitätsmedizin Berlin, Germany we included minors from 15 years of age onwards if they gave written consent. Regarding minors below 15 years of age we additionally obtained the consent of their present legal guardian. Cases were consecutively enrolled until the required sample size was obtained. For each case, two controls were enrolled on the same day.

### Data collection and statistical analysis

Data collection was performed in face-to-face one-hour interviews based on a 66-item questionnaire conducted by four trained local midwives. The questionnaire had been developed exclusively for our study purpose and setting and was adjusted after a test period of one week. Information collected through the questionnaire covered sociodemographic, obstetric, medical, nutritional, environmental, and behavioral characteristics as well as diagnostic information and birth outcomes (see Additional Files 1 and 2). Sociodemographic characteristics included age, religion, ethnicity, relationship status, education, occupation, maternal assets, place of residence, travel time and costs to hospital, number of children, and economic status. The economic status was assessed with a nine-point score. Obstetric characteristics asked for were gravidity, previous spontaneous abortions, and stillbirths as well as induced abortions, interpregnancy interval, multifetal gestation, first pregnancy with current partner, an obstetric history of PrE/E, family predisposition for PrE/E, and the number of antenatal care (ANC) visits. Medical characteristics assessed were information on prolonged diarrhoea defined as a duration of ≥2 weeks, UTI, anemia, gestational diabetes, placenta previa, and Malaria during pregnancy. Furthermore, we acquired information about preexisting hypertension, family predisposition of hypertension, pregestational and gestational diabetes, and family predisposition of diabetes as well as constitutional variables like body weight, and visual evidence of overweight. We limited family predisposition on first-degree relatives meaning mother, father, or full siblings. Preexisting hypertension was defined as high blood pressure diagnosed before the 20th gestational week. The MUAC was measured by study nurses on the woman’s mid-upper left arm (the right arm if she was left-handed). We defined an obese pre-pregnancy body size with a MUAC > 32 cm relating to Okereke et al., who defined a MUAC of 33 cm as a reliable cut-off point for obesity in Nigeria [33]. The visual evidence of overweight described a subjective appraisal of the attending study nurse regarding the excess bodyweight of the participant. Nutritional characteristics assessed were calcium sources in the daily diet as well as the frequency of intake of fruits, vegetables, meat, and animal products, which we categorized as an adequate intake defined as weekly or daily or an inadequate intake defined as every 2 weeks, monthly or never. Environmental characteristics included sources of pollution like living close to a main road, waste deposit, oil refinery, or cooking with open fire in a closed room, working with chemicals or other sources. We also assessed the source of which women mainly drew their drinking water regarding the poorly managed water supply system in Sierra Leone [34]. Behavioral characteristics consisted of consumption of alcohol, smoking behavior, heavy physical work during pregnancy, sleep duration and quality, emotional stress, as well as visits to a traditional healer and receiving traditional treatment. There was no follow-up of study participants, except for retrieving data of subsequent birth outcomes among the pregnant cases from available patient charts. Birth outcomes included low birth weight defined as below 2500 g, and preterm birth was defined as a fetus born prior to the 37th postmenstrual week. Questionnaires were stored in a safe in PCMH which was only accessible for one member of the study team. All data were entered into an Excel Database in anonymized form and continuously crosschecked. IBM SPSS statistical software package version 25.0 (IBM, Armonk, NY, USA) was used to perform the statistical analysis. Categorical variables were expressed as frequencies with percentages. Metric variables were expressed as mean with standard deviation in case of a normal distribution or median and interquartile ranges in case of skewed distribution. For univariate analysis with categorical variables, we used Pearson’s Chi-square Test. Metric variables were compared using Independent T-Test and Mann-Whitney U-Test. Binary logistic regression analysis was performed to identify independent risk factors. Criteria for inclusion were a *p*-value ≤0.001 and odds ratio > 1.5 in univariate analysis. Variables that were additionally considered with a p-value < 0.05 were relationship status, first pregnancy with current partner, animal product intake, snoring, and Malaria. We additionally included Malaria as a risk factor because of the previously described strong influence of *Plasmodium falciparum*-infected placenta on PrE/E [19–21]. We did not consider relationship status, animal product intake, and snoring as that variables did not prove to have a significant influence in previous literature. Regarding the variable first pregnancy with current partner, we did not take it into account because of the potential risk of being confounded by for example the age of the woman [35]. Before analysis, questions with a very low response rate of ≥50% of missing values like current weight, smoking behavior, alcohol consumption, calcium sources in the daily diet as well as family predisposition of diabetes and pregestational diabetes were excluded. We tried to complete variables like gestational diabetes, anemia during pregnancy, and placenta previa with patient charts which turned out to be not reliable due to lacking data entries. Further variables with missing data were handled by employing multiple imputation to include all 672 study participants into a binary logistic regression model. After analyzing the missing data patterns of 47 eligible variables, we identified 23 variables that had ≥1% missing data. We excluded two variables, ‘interpregnancy interval’ and ‘obstetric history of PrE/E’ because missing data exceeded 30% due to a high rate of nulliparous women in our sample. As there were no patterns in the missing data of the remaining 21 variables, we chose them for imputation. Variable chosen for imputation in descending order of quantity of missing values were family predisposition for PrE/E of all female family members, family predisposition for PrE/E of only mother and sister, travel cost, family predisposition for chronic hypertension, living close to a main road**,** living close to a waste deposit, environmental pollution, maternal assets, travel time, average sleep duration, meat intake**,** place of residence, occupation, first pregnancy with current partner, vegetable intake, fruit intake, parity, MUAC, well or borehole water as main source of drinking water, snoring and daytime sleepiness. We added four additional variables that had < 1% of missing values but were chosen to be included in the binary logistic regression model which were Malaria, prolonged diarrhoea**,** UTI, and preexisting hypertension**.** We imputed 25 variables with 10 datasets, which raised the availability of datasets for calculation from 65.5% (440) to 100% (672). For imputation, we chose the Mersenne Twister as a random number generator program and for generator initialization, we chose the fixed value of 2,000,000. Regarding data distribution, the imputed data set showed an overall good validity. Statistical significance was set at *p*-value < 0.05, crude and adjusted odds ratios (COR, AOR) and 95% confidence interval (95% CI) were calculated. The Hosmer-Lemeshow goodness of fit test for logistic regression was applied and showed overall a good fit with a *p*-value > 0.05.

## Results

A total of 697 women, 233 cases and 464 controls were recruited among pregnant and postpartum women in PCMH**.** Before analysis, we had to exclude 25 women who did not meet the inclusion criteria,19 cases and 6 controls. 19 cases were falsely diagnosed with PrE/E with 17 women who did not meet national guidelines of PrE/E diagnosis because the blood pressure assessed in one reading was below 140/90 mmHg. One woman had complete missing diagnosis data and an eclamptic case subsequently proved to be epileptic. Out of 6 controls, 4 had episodes of gestational hypertension in pregnancy with suspected preeclampsia and 2 had an abortion before 20 weeks of pregnancy. We analyzed data from 672 women, 214 cases, and 458 controls. In the case group, 63 (29.4%) women were eclamptic and two died after participation.

### Sociodemographic characteristics

Women were on a mean average 25 years old, had one child, and lived by a majority of 78% in a relationship. They mainly belonged to the Muslim religion (70.8%) and the Temne tribe (40.1%). Almost half of the cohort were illiterate (45.1%) and up to 20% were adolescents including 7 minors. Most women worked as self-employed traders (46.5%) and twice as many cases (31.6%) were housewives compared to controls (14.1). Among postpartum recruited women, we observed an average of 5 ANC visits during their pregnancy. Women in the case group dwelled more often in a poorly equipped household (COR = 1.92, 95% CI: 1.18–3.14) without the availability of electricity (COR = 1.72, 95% CI: 1.20–2.45) and pipe-borne water (COR = 1.47, 95% CI: 1.06–2.04). In the case group, 40.0% of women had less than 50,000 Sierra Leone Leones (SLL) of funds available per month, which equals about less than 5 US-Dollar (COR = 2.72, 95% CI: 1.89–3.92). Sociodemographic differences between cases and controls are fully displayed in Table 1.

**Table 1:** Sociodemographic characteristics of women with and without PrE/E in PCMH

Characteristics	Total n (%)	Cases n (%)	Controls n (%)	COR (95% CI)	*p*-value
Total	672	214	458		
Age	(668)	(213)	(455)		
Means	25.30 (±5.19)	25.01 (± 5.95)	25.43 (± 5.91)		0.401
< 20	128 (19.2)	47 (22.1)	81 (17.8)	1.33 (0.88–2.02)	0.171
20–32	442 (66.2)	134 (62.9)	308 (67.7)	1.0	
> 32	98 (14.7)	32 (15.0)	66 (14.5)	1.11 (0.70–1.78)	0.650
Relationship^a^	(668)	(211)	(457)		
Yes	521 (78.0)	175 (82.9)	346 (75.7)	1.56 (1.03–2.37)	0.036
No	147 (22.0)	36 (17.1)	111 (24.3)	1.0	
Religion	(662)	(212)	(450)		
Christian	193 (29.2)	59 (27.8)	134 (29.8)	1.0	
Muslim	469 (70.8)	153 (72.2)	316 (70.2)	1.10 (0.77–1.58)	0.607
Ethnicity	(669)	(212)	(457)		
Mende	104 (15.5)	29 (13.7)	75 (16.4)	1.0	
Temne	268 (40.1)	89 (42.0)	179 (39.2)	1.29 (0.78–2.12)	0.322
Other^b^	297 (44.4)	94 (44.3)	203 (44.4)	1.20 (0.73–1.96)	0.474
Education	(669)	(212)	(457)		
Illiterate	302 (45.1)	104 (49.1)	198 (43.3)	1.26 (0.91–1.75)	0.166
literate	367 (54.9)	108 (50.9)	259 (56.7)	1.0	
Occupation	(662)	(209)	(453)		
Housewife	130 (19.6)	66 (31.6)	64 (14.1)	3.88 (2.43–6.22)	< 0.001
Trader	308 (46.5)	96 (45.9)	212 (46.8)	1.71 (1.14–2.55)	0.009
Other^c^	224 (33.8)	47 (22.5)	177 (39.1)	1.00	
Number of Children	(672)	(214)	(458)		
Own children	1.01 (±1.20)	1.08 (±1.24)	0.98 (±1.17)		0.307
Other children	0.33 (±0.71)	0.27(±0.68)	0.36 (±0.72)		0.115
Place of residence	(657)	(207)	(450)		
City	487 (74.1)	151 (72.9)	336 (74.7)	1.0	
Suburb/Rural	170 (25.9)	56 (27.1)	114 (25.3)	1.09 (0.75–1.59)	0.640
Antenatal care visits^d^	(672) 5.36 (±2.61)	(214) 5.76 (±2.34)	(458) 5.18 (±2.70)		
Travel time^e^	(647)	(208)	(439)		
Median	45 (30–60)	60 (41–90)	40 (30–60)		< 0.001
Means	52 (± 34.91)	69 (± 38.63)	45 (± 30.02)		
Travel cost^f^	(540)	(194)	(346)		
Median	3000 (2000–6000)	5000 (3000–8000)	2750 (2000–4075)		< 0.001
Means	5600 (±8800)	7600 (±10,400)	4500 (±7600)		
Maternal assets^g^	(647)	(205)	(442)		
≤ 50,000 SLL	169 (26.1)	82 (40.0)	87 (19.7)	2.72 (1.89–3.92)	< 0.001
> 50,000 SLL	478 (73.9)	123 (60.0)	355 (80.3)	1.0	
Economic status^h^	(668)	(211)	(457)		
Low	184 (27.5)	75 (35.5)	109 (23.9)	1.92 (1.18–3.14)	0.008
Middle	355 (53.1)	102 (48.3)	253 (55.4)	1.13 (0.72–1.77)	0.607
High	129 (19.3)	34 (16.1)	95 (20.8)	1.0	
Pipe-borne water	(667)	(210)	(457)		
Yes	377 (56.5)	105 (50.0)	272 (59.5)	1.0	
No	290 (43.5)	105 (50.0)	185 (40.5)	1.47 (1.06–2.04)	0.021
Electricity	(667)	(210)	(457)		
Yes	490 (73.4)	139 (65.9)	351 (76.8)	1.0	
No	178 (26.6)	72 (34.1)	106 (23.2)	1.72 (1.20–2.45)	0.003

Univariate analysis was conducted with Pearson’s Chi-square test for categorical variables. Metric variables were compared using Independent T-Test and Mann-Whitney-U-Test. Statistical significance was set at *p*-value < 0.05, crude and adjusted odds ratios and 95% confidence interval were calculated

^a^ relationship status: yes = cohabit, married; no = single, divorced, widowed

^b^ other ethnicities = Limba, Krio, Fullah, other

^c^ other occupation = farmer, student, other

^d^ excluded from univariate analysis because the variable is confounded by pregnancy status during recruitment

^e^ to Princess Christian Maternity Hospital in minutes

^f^ to Princess Christian Maternity Hospital in Sierra Leone Leones

^g^ monthly assets; 50,000 Sierra Leone Leones equal about 5 US-Dollar

^h^ economic status: electricity, radio, cattle, pipe-borne water, TV, motorbike or car, mobile phone, fridge, mosquito-net; score: low = 0–3, middle = 4–6, high = 7–9

### Manifestations of PrE/E

The typical symptomatic manifestation of preeclampsia in our study included headache (96.0%), generalized edema (82.0%), and epigastric pain (78.0%). The majority of eclamptic cases suffered from vomiting (77.4%) and blurred vision (67.7%). Cases had a mean blood pressure of 161/109 mmHg, and 2.5+ proteinuria in urinalysis.

### Univariate analysis of risk factors

Women with PrE/E in a previous pregnancy had an almost threefold risk for PrE/E (COR = 2.62, 95% CI: 1.12–6.10), while family predisposition for PrE/E in the mother or sister even led to a fivefold risk compared to women without a family history or PrE/E (COR = 5.33, 95% CI: 2.66–10.69). Preexisting hypertension was significantly more often found in the case group with 7.2% of women compared to only 2.0% of women in the control group (COR = 3.88, 95% CI: 1.67–9.01). However, family predisposition for hypertension had no significant impact on the risk of PrE/E. To have the first pregnancy with the current partner was also significantly connected to PrE/E (COR = 1.68, 95% CI: 1.17–2.41). Medical conditions like UTI (COR = 2.66, 95% CI: 1.82–3.88), prolonged diarrhoea (COR = 3.71, 95% CI: 2.49–5.53), and Malaria (COR = 1.60, 95% CI: 1.13–2.27) also significantly increased the risk for PrE/E. In the case group, more women appeared visually to be overweight (16.4%) compared to the control group (2.0%). In accordance with that, a high MUAC > 32 cm was measured in 23.9% of the case group and only 10.5% of the control group (COR = 2.67, 95% CI: 1.73–4.14). Women in the case group proved to have a significantly lower intake of fruits (COR = 3.30, 95% CI: 2.25–4.85) as well as animal products other than meat, while inadequate vegetable intake seemed to have a protective effect (COR = 0.65, 95% CI: 0.47–0.90). A short sleep duration of < 6 h was significantly more often observed in the case group (COR = 1.94, 95% CI: 1.36–2.77), as well as snoring (COR = 1.82, 95% CI: 1.18–2.79). About 65% of women in our study cohort stated that they were affected by pollution in their daily living environment. In the case group, nearly twice as many women lived close to a waste deposit (29.4%) compared to the control group (16.3%) (COR = 2.14, 95% CI: 1.44–3.18) and more cases used well or borehole water as their main source of drinking water (COR = 1.90, 95% CI: 1.30–2.79) (Table 2).

**Table 2:** Obstetrics, medical, nutritional, environmental and behavioural characteristics of women with and without PrE/E in PCMH

Characteristics	Total n (%)	Cases n (%)	Controls n (%)	COR (95% CI)	*p*-value
**Gravidity**	(672)	(214)	(458)		
1	244 (36.3)	84 (39.3)	160 (34.9)	1.62 (1.02–2.58)	0.041
2	143 (21.3)	35 (16.4)	108 (23.6)	1.0	
> 2	285 (42.4)	95 (44.4)	190 (41.5)	1.54 (0.98–2.43)	0.060
**Parity**	(664)	(212)	(452)		
0	258 (38.9)	87 (41.0)	171 (37.8)	1.14 (0.82–1.60)	0.429
≥ 1	406 (61.1)	125 (59.0)	281 (62.2)	1.0	
**Previous spontaneous abort or stillbirth**	(669)	(212)	(457)		
Yes	69 (10.3)	17 (8.0)	52 (11.4)	0.68 (0.38–1.21)	0.184
No	600 (89.7)	195 (92.0)	405 (88.6)	1.0	
**Previous induced abortion**	(671)	(214)	(457)		
Yes	46 (6.9)	9 (4.2)	37 (8.1)	0.50 (0.24–1.05)	0.063
No	625 (93.1)	205 (95.8)	420 (91.9)	1.0	
**Interpregnancy interval**	(411)	(124)	(287)		
≤ 18 months	35 (8.5)	13 (10.5)	22 (7.7)	1.45 (0.70–2.99)	0.311
18–120 months	349 (84.9)	101 (81.5)	248 (86.4)	1.0	
> 120 months	27 (6.6)	10 (8.1)	17 (5.9)	1.44 (0.64–3.26)	0.374
**Multifetal gestation**	(671)	(213)	(458)		
Yes	45 (6.7)	18 (8.5)	27 (5.9)	1.47 (0.79–2.74)	0.218
No	626 (93.3)	195 (91.5)	431 (94.1)	1.0	
**First pregnancy with current partner**	(662)	(211)	(451)		
Yes	443 (66.9)	157 (74.4)	286 (63.4)	1.68 (1.17–2.41)	0.005
No	219 (33.1)	54 (25.6)	165 (36.6)	1.0	
**Preeclampsia or eclampsia in obstetric history**	(407)	(125)	(282)		
Yes	23 (5.7)	12 (9.6)	11 (3.9)	2.62 (1.12–6.10)	0.022
No	384 (94.3)	113 (90.4)	271 (96.1)	1.0	
**Family predisposition for preeclampsia and eclampsia**	(525)	(138)	(387)		
Yes	37 (7.0)	23 (16.7)	14 (3.6)	5.33 (2.66–10.69)	< 0.001
No	488 (93.0)	115 (83.3)	373 (96.4)	1.0	
**Preexisting hypertension**	(666)	(208)	(458)		
Yes	24 (3.6)	15 (7.2)	9 (2.0)	3.88 (1.67–9.01)	0.001
No	642 (96.4)	193 (92.8)	449 (98.0)	1.0	
**Family predisposition for chronic hypertension**	(612)	(178)	(434)		
Yes	113 (18.5)	35 (19.7)	78 (18.0)	1.12 (0.72–1.74)	0.624
No	499 (81.5)	143 (80.3)	356 (82.0)	1.0	
**Prolonged diarrhoea**	(671)	(214)	(457)		
Yes	127 (18.9)	72 (33.6)	55 (12.0)	3.71 (2.49–5.53)	< 0.001
No	544 (81.1)	142 (66.4)	402 (88.0)	1.0	
**UTI**	(670)	(214)	(456)		
Yes	145 (21.6)	72 (33.6)	73 (16.0)	2.66 (1.82–3.88)	< 0.001
No	525 (78.4)	142 (66.4)	383 (84.0)	1.0	
**Malaria**	(671)	(214)	(457)		
Yes	432 (64.4)	153 (71.5)	279 (61.1)	1.60 (1.13–2.27)	0.008
No	239 (35.6)	61 (28.5)	178 (38.9)	1.0	
**Visual evidence of overweight**	(671)	(213)	(458)		
Yes	44 (6.6)	35 (16.4)	9 (2.0)	9.95 (4.67–21.19)	< 0.001
No	548 (81.7)	154 (72.3)	394 (86.0)	1.0	
Don’t know	79 (11.8)	24 (11.3)	55 (12.0)		
**MUAC**	(665)	(209)	(456)		
> 32 cm	98 (14.7)	50 (23.9)	48 (10.5)	2.67 (1.73–4.14)	< 0.001
≤ 32 cm	567 (85.3)	159 (76.1)	408 (89.5)	1.0	
**Fruit intake**	(664)	(209)	(455)		
Inadequate	141 (21.2)	75 (35.9)	66 (14.5)	3.30 (2.25–4.85)	< 0.001
Adequate	523 (78.8)	134 (64.1)	389 (85.5)	1.0	
**Vegetable intake**	(664)	(211)	(453)		
Inadequate	326 (49.1)	88 (41.7)	238 (52.5)	0.65 (0.47–0.90)	0.009
Adequate	338 (50.9)	123 (58.3)	215 (47.5)	1.0	
**Meat intake**	(656)	(211)	(445)		
Inadequate	427 (65.1)	147 (69.7)	280 (62.9)	1.35 (0.95–1.92)	0.090
Adequate	229 (34.9)	64 (30.3)	165 (37.1)	1.0	
**Animal product intake**	(667)	(211)	(456)		
Inadequate	253 (37.9)	92 (43.6)	161 (35.3)	1.42 (1.02–1.98)	0.040
Adequate	414 (62.1)	119 (56.4)	295 (64.7)	1.0	
**Environmental pollution**^a^	(631)	(201)	(430)		
Yes	408 (64.7)	154 (76.6)	254 (59.1)	2.27 (1.55–3.32)	< 0.001
No	223 (35.3)	47 (23.4)	176 (40.9)	1.0	
**Living close to a waste deposit**	(631)	(201)	(430)		
Yes	129 (20.4)	59 (29.4)	70 (16.3)	2.14 (1.44–3.18)	< 0.001
No	502 (79.6)	142 (70.6)	360 (83.7)	1.0	
**Living close to a main road**	(631)	(201)	(430)		
Yes	257 (40.7)	80 (39.8)	177 (41.2)	0.95 (0.67–1.33)	0.746
No	374 (59.3)	121 (60.2)	253 (58.8)	1.0	
**Well or borehole water as main source of drinking water**	(665)	(211)	(454)		
Yes	141 (21.2)	61 (28.9)	80 (17.6)	1.90 (1.30–2.79)	0.001
No	524 (78.8)	150 (71.1)	374 (82.4)	1.0	
**Average sleep duration**	(648)	(198)	(450)		
Short < 6 h	386 (59.6)	139 (70.2)	247 (54.9)	1.94 (1,36–2.77)	< 0.001
Long ≥6 h	262 (40.4)	59 (29.8)	203 (45.1)	1.0	
**Snoring**	(665)	(211)	(454)		
Yes	104 (15.6)	45 (21.3)	59 (13.0)	1.82 (1.18–2.79)	0.006
No	561 (84.4)	166 (78.7)	395 (87.0)	1.0	
**Heavy physical work**^b^	(671)	(214)	(457)		
Yes	411 (61.3)	113 (52.8)	298 (65.2)	0.60 (0.43–0.83)	0.002
No	260 (38.7)	101 (47.2)	159 (34.8)	1.0	
**Emotional stress**	(666)	(212)	(454)		
Yes	234 (35.1)	76 (35.8)	158 (34.8)	0.96 (0.68–1.34)	0.792
No	432 (64.9)	136 (64.2)	296 (65.2)	1.0	
**Visit of traditional healer**	(667)	(214)	(453)		
Never	520 (78.0)	167 (78.0)	353 (77.9)	1.0	0.705
Sometimes	136 (20.4)	46 (21.5)	90 (19.9)	1.08 (0.72–1.61)	0.104
Often	11 (1.6)	1 (0.5)	10 (2.2)	0.21 (0.03–1.67)	
**Traditional treatment**	(667)	(214)	(453)		
Never	462 (69.3)	149 (69.6)	313 (69.1)	1.0	
Sometimes	174 (26.1)	63 (29.4)	111 (24.5)	1.19 (0.83–1.72)	0.345
Often	31 (4.6)	2 (0.9)	29 (6.4)	0.15 (0.03–0.62)	0.003

Univariate analysis was conducted with Pearson’s Chi-square test for categorical variables. Metric variables were compared using Independent T-Test and Mann-Whitney-U-Test. Statistical significance was set at *p*-value < 0.05, crude and adjusted odds ratios and 95% confidence interval were calculated

^a^ environmental pollution of daily living environment: close to waste deposit, close to main road, cooking with open fire in closed room, working with chemicals e.g. pesticides, paint or car components, close to oil refinery, other sources of pollution

^b^ heavy physical work: carry heavy weights, farm work, long distance walking (≥2 h per day), prolonged standing (≥6 h per day), manual laundry, carry heavy weights on head, other physical work

### Multivariate analysis of independent risk factors for PrE/E

Logistic regression analysis, as shown in Table 3, revealed nine independent predictors for PrE/E: a family predisposition for PrE/E (AOR = 2.72, 95% CI: 1.46–5.07), preexisting hypertension (AOR = 3.64, 95% CI: 1.32–10.06), a high MUAC (AOR = 3.09, 95% CI: 1.83–5.22), presence of UTI during pregnancy (AOR = 2.02, 95% CI: 1.28–3.19), presence of prolonged diarrhoea during pregnancy (AOR = 2.81, 95% CI: 1.63–4.86), low maternal assets (AOR = 2.56, 95% CI: 1.63–4.02), inadequate fruit intake (AOR = 2.58, 95% CI: 1.64–4.06), the use of well or borehole water as the main source of drinking water (AOR = 2.05, 95% CI: 1.31–3.23) as well as living close to a waste deposit (AOR = 1.94, 95% CI: 1.15–3.25). From all analyzed factors, only Malaria (AOR = 1.43, 95% CI: 0.95–2.15) and short sleep duration (AOR = 1.48, 95% CI: 0.96–2.29) were not independently associated with PrE/E.

**Table 3:** Binary logistic regression model of predictors for PrE/E in PCMH

Characteristics	Case n (%)	Control n (%)	COR (95% CI)	*p*-value	AOR (95%CI)	*p*-value
**Maternal assets**						
≤ 50,000 SLL	82 (40.0)	87 (19.7)	2.72 (1.89–3.92)	< 0.001	2.56 (1.63–4.02)	<
> 50,000 SLL	123 (60.0)	355 (80.3)	1.0		1.0	0.001
**Family predisposition for preeclampsia and eclampsia**						
Yes	23 (16.7)	14 (3.6)	5.33 (2.66–10.69)	< 0.001	2.72 (1.46–5.07)	0.002
No	115 (83.3)	373 (96.4)	1.0		1.0	
**Preexisting hypertension**						
Yes	15 (7.2)	9 (2.0)	3.88 (1.67–9.01)	0.001	3.64 (1.32–10.06)	0.013
No	193 (92.8)	449 (98.0)	1.0		1.0	
**UTI**						
Yes	72 (33.6)	73 (16.0)	2.66 (1.82–3.88)	< 0.001	2.02 (1.28–3.19)	0.002
No	142 (66.4)	383 (84.0)	1.0		1.0	
**Prolonged diarrhoea**						
Yes	72 (33.6)	55 (12.0)	3.71 (2.49–5.53)	< 0.001	2.81 (1.63–4.86)	< 0.001
No	142 (66.4)	402 (88.0)	1.0		1.0	
**Malaria**						
Yes	153 (71.5)	279 (61.1)	1.60 (1.13–2.27)	0.008	1.43 (0.95–2.15)	0.090
No	61 (28.5)	178 (38.9)	1.0		1.0	
**MUAC**						
> 32 cm	50 (23.9)	48 (10.5)	2.67 (1.73–4.14)	< 0.001	3.09 (1.83–5.22)	< 0.001
≤ 32 cm	159 (76.1)	408 (89.5)	1.0		1.0	
**Living close to a waste deposit**						
Yes	59 (29.4)	70 (16.3)	2.14 (1.44–3.18)	< 0.001	1.94 (1.15–3.25)	0.013
No	142 (70.6)	360 (83.7)	1.0		1.0	
**Well or borehole water as main source of drinking water**						
Yes	61 (28.9)	80 (17.6)	1.90 (1.30–2.79)	0.001	2.05 (1.31–3.23)	0.002
No	150 (71.1)	374 (82.4)	1.0		1.0	
**Average sleep duration**						
Short < 6 h	139 (70.2)	247 (54.9)	1.94 (1,36–2.77)	< 0.001	1.48 (0.96–2.29)	0.078
Long ≥6 h	59 (29.8)	203 (45.1)	1.0		1.0	
**Fruit intake**						
Inadequate	75 (35.9)	66 (14.5)	3.30 (2.25–4.85)	< 0.001	2.58 (1.64–4.06)	< 0.001
Adequate	134 (64.1)	389 (85.5)	1.0		1.0	

Binary Logistic Regression Model was calculated including variables matching criteria of a *p*-value ≤0.001 and crude odds ratios > 1.5. We additionally included the risk factor Malaria during pregnancy because of its previously described influence of *Plasmodium falciparum* infected placenta on PrE/E. Multiple Imputation was conducted with the Mersenne Twister as random number generator program and for generator initialization we chose the fixed value of 2,000,000. We imputed 25 variables with 10 datasets. Statistical significance was set at *p*-value < 0.05, crude and adjusted odds ratios and 95% confidence interval were calculated. The Hosmer Lemeshow goodness of fit test for logistic regression was applied and showed overall a good fit with a *p*-value > 0.05

### Birth outcomes

Adverse birth outcomes linked to PrE/E were preterm birth (COR = 4.72, CI95%: 3.12–7.16), low birth weight of neonates (COR = 4.67, CI95%: 2.81–7.76) and macerated stillbirths (COR = 2.81, CI95%: 1.33–5.97). However, the overall neonatal mortality rate was not significantly higher in the case group (14.2%) compared to the control group (9.7%) (Table 4).

**Table 4:** Birth outcomes in PCMH

Characteristics	Total n (%)	Case Births n (%)	Control Births n (%)	COR (95% CI)	*p*-value
**Total**	713	229	484		
**Gestational age at delivery** ^**a**^	599	153	446		
Median	38 (37–40)	37 (36–39)	38 (37–40)		
Means	37.91 (± 2.78)	36.90 (± 3.36)	38.26 (± 2.46)		< 0.001
**Birth outcome**	(653)	(169)	(484)		
Live birth	582 (89.1)	145 (85.8)	437 (90.3)	1.0	
Fresh stillbirth	42 (6.4)	10 (5.9)	32 (6.6)	0.94 (0.45–1.96)	0.873
Macerated stillbirth	29 (4.4)	14 (8.3)	15 (3.1)	2.81 (1.33–5.97)	0.005
**Delivery mode**	(653)	(169)	(484)		
Vaginal spontaneous	465 (71.2)	113 (66.9)	352 (72.7)	1.0	
Vaginal operative	39 (6.0)	4 (2.4)	35 (7.2)	0.36 (0.12–1.02)	0.046
Caesarean section	149 (22.8)	52 (30.8)	97 (20.0)	1.67 (1.12–2.49)	0.011
**Preterm birth** ^b^	(598)	(153)	(445)		
Yes	130 (21.7)	67 (43.8)	63 (14.3)	4.72 (3.12–7.16)	< 0.001
No	468 (78.3)	86 (56.2)	382 (85.7)	1.0	
**Birth weight** ^c^	(543)	(132)	(411)		
< 2500 g	75 (13.8)	40 (30.3)	35 (8.5)	4.67 (2.81–7.76)	< 0.001
≥ 2500 g	468 (86.2)	92 (69.7)	376 (91.5)	1.0	

Univariate analysis was conducted with Pearson’s Chi-square test for categorical variables. Metric variables were compared using Independent T-Test and Mann-Whitney-U-Test. Statistical significance was set at *p*-value < 0.05, crude and adjusted odds ratios and 95% confidence interval were calculated

^a^ gestational age at delivery; caesarean section included

^b^ fetus born prior to the 37th postmenstrual week; caesarean section included

^c^ multifetal gestation excluded; caesarean section included

## Discussion

Our study represents the first comprehensive assessment of risk factors for PrE/E in Sierra Leone. Considering the scarcity of data in our setting, we analyzed a wide spectrum of factors potentially influencing pregnant women’s health. Our main findings included a preexisting hypertension, a high MUAC, presence of UTI or prolonged diarrhoea during pregnancy, family predisposition for PrE/E, inadequate fruit intake, and low maternal assets as independent risk factors for PrE/E. In addition, we explored the influence of environmental pollution on pregnant women’s health and found that living close to a waste deposit as well as using well or borehole water as main source of drinking water were independently linked with PrE/E. Among those identified factors, preexisting hypertension had the strongest association with PrE/E with an approximate fourfold increased risk. This finding corroborates a secondary analysis of a WHO Global Survey conducted in 23 low and middle-income countries, which reported that women who suffered chronic hypertension were almost 8 times more likely to become preeclamptic or eclamptic than women without the condition [9]. Chronic hypertension is linked to vascular dysfunction, which is assumed to play a key role in the pathophysiology of PrE/E by inducing abnormal development of placental vasculature in early pregnancy, leading to disordered placental perfusion [36]. In this context, obesity is another important player, additionally fostering high blood pressure and inducing cardiovascular morbidity, which hence promotes PrE/E [36]. Obese study participants from our cohort with a MUAC > 32 cm showed a threefold increased risk for PrE/E. This is supported by research from Zimbabwe, where a MUAC ≥28 cm already increased the risk for preeclampsia about 4.4 times [26], and Endeshaw et al. (2015) reported that in Ethiopia the incidence rate of preeclampsia raised by a factor of 1.35 with every 1 cm increase of MUAC [23]. UTIs and prolonged diarrhoea during pregnancy both doubled the risk for PrE/E in our cohort. A systematic review investigating the association between maternal infection and preeclampsia showed that women suffering from UTIs during pregnancy were 57% more likely to develop the condition [10]. Von Dadelszen et al. (2002) suggest that inflammatory states can induce acute atherosis, causing uteroplacental perfusion insufficiency [37]. These results are consistent with findings from Bilano et al. and Kaduma et al., who reported that preeclamptic women in Tanzania were 7.7 times more likely to present with significant bacteriuria [9, 38]. A heritable component with a family predisposition of PrE/E in the mother or sister of a woman proved to be independently associated with a threefold higher risk for PrE/E in our analysis. In a multi-generation study accessing a birth register including 32,824 preeclamptic women, Nilsson et al. stated that a family predisposition for preeclampsia in a full-sister or mother raised a woman’s risk about two to threefold, whereby an affected half-sister had no significant influence on the risk of a woman [8]. Assessing unmodifiable risk factors like the family predisposition of a pregnant woman should be a priority in the first ANC visit and enable women at risk to get access to closer monitoring and education on early signs and symptoms of PrE/E. With this study being situated in the urban setting of Freetown, 65% of women in our cohort stated to be affected by environmental pollution; living close to a waste deposit doubled the risk for PrE/E in our setting. Two recent studies from the DRC and a study from Nigeria emphasize that heavy metal pollution could raise the risk for preeclampsia; in those studies, preeclamptic women had significantly higher blood lead levels as well as urinary lead extraction [14–16]. In this regard, a meta-analysis from 2018 concluded that the likelihood of a woman becoming preeclamptic increased by about 1.6% with every 1 μg/dL rise in their blood lead concentration [39]. Frequent monitoring of blood lead levels in pregnant women is unrealistic for our setting, but further research is needed to have a better understanding of the sources of pollution in Freetown. Beyond that, significantly more preeclamptic and eclamptic women drew their drinking water from a well or borehole, which doubled their risk for PrE/E. In 2019, WHO and United Nations Children’s Fund published a report stating that in Sierra Leone only 10% of the population had access to ‘safely managed’ drinking water services, defined as located inside the yard, available for at least 12 h per day and in line with standards regarding fecal and chemical contamination [34]. Nutritional factors also played an important role in our study, as an inadequate intake of fruits raised the risk for PrE/E almost threefold. In accordance, several studies from Ethiopia described a regular intake of fruits and vegetables to be protective against preeclampsia [22–25]. Interestingly, in contrast to those findings, the consumption of vegetables less than one time per week proved to be protective for PrE/E in our analysis. This may again hint to environmental pollution exposure, following findings by Kim et al. (2016), who found vegetables to be one of the most lead loaded aliments affecting maternal health in the Republic of Korea [40]. Almost all of the aforementioned risk factors could be controlled through regular and frequent ANC visits with adequate screening and counseling. As a matter of fact, the National Protocols and Guidelines for Emergency Obstetric and Newborn Care recommend measuring blood pressure or performing urinalysis as well as counseling on nutrition, diet, and exercise at every single ANC visit [41]. However, in our sample, the average number of ANC visits until birth was far below the eight visits recommended by the National Protocols and Guidelines. One reason could be a general mistrust among pregnant women in health care facilities in Sierra Leone. This is mirrored in a recent qualitative study by Theuring et al. (2018) in the same hospital setting, which described some reluctance among pregnant women towards facility-based delivery care, suggesting that PCMH might need to improve communication skills of staff to build a trustful environment for their clients [42]. Sripad et al. (2019) supports this finding in their study from Nigeria, stating that respectful maternity care affects the willingness to seek care in ANC clients [43]. Above that, ANC services should be revised to offer more effective management of PrE/E with extended risk factor assessment and management as well as education on early signs and symptoms of PrE/E and if necessary, more frequent screenings for the condition. Regarding risk factor assessment and management, the skills of ANC staff might need to be widened towards a comprehensive strategy, including not only systematic screening of physical risk factors, but also intense counseling. This should comprise behavioral risk factors, like hygiene-related behavior to avoid infectious diseases presenting in UTIs or prolonged diarrhoea, and appropriate nutrition and diet counseling, including education on drinking water management. In this context, it would be worthwhile to focus on practical information empowering particularly those women lacking financial resources, for example by advising them how to cultivate fruits at home to improve their diet or teaching them how to obtain safe drinking water by boiling it and storing it correctly. The great poverty of the women in our setting must be apprehended as an underlying factor not only for low access to nutritious food and safe water supply but also for most other risk factors, including housing close to sources of pollution. In the case group, 40% of women had less than 50,000 SLL per month available to spend. In Nigeria, Sripad et al. found that the financial situation of women imposed a barrier of care-seeking in the early stages of PrE/E [43]. Health policymakers in Sierra Leone should, therefore, take into serious consideration how women with an identified high risk for a condition like PrE/E could benefit from reduced costs on healthy food, drinking water supply, and transportation to access healthcare services regularly, to enable ANC services to properly screen and manage clients at risk for PrE/E.

### Limitations

The facility-based study design might entail a selection bias in the recruitment of the control group, because women who sought skilled delivery services or received inpatient treatment in the referral institution PCMH are likely to have experienced a more complicated pregnancy, e.g. with higher rates of multifetal gestations or with more severe complications during the current or previous pregnancy. Other limitations were low educational levels of participants, reduced diagnostic capacities due to the resource-limited setting, and lacking patient documentation. As a result, we had to face missing data as well as underreporting of e.g. reasons for previous pregnancy complications resulting in abortions or stillbirth as well as provided history of preexisting conditions.

## Conclusions

PrE/E is one of the leading causes of maternal mortality in Sierra Leone. Our findings suggest that systematic assessment of identified PrE/E risk factors, including a family history of PrE/E, preexisting hypertension, or obesity by measuring the MUAC, should be performed early on in ANC, followed by continued close monitoring of potential behavioral risk factors throughout pregnancy to improve patient management. Counseling on nutrition, dietary aspects and exercise should be comprehensively addressed and be extended with practical information meaningful for the poorest parts of the population, such as on self-cultivation of fruits and achieving improved water safety. Regarding the high prevalence of UTIs and prolonged diarrhoea, improved hygiene-related behavior should also be strongly emphasized. Additionally, our study implies that further research on sources of environmental pollution in Freetown, especially regarding heavy metals like lead strain, is urgently needed.

## Supplementary Information

**Additional file 1.** CASE GROUP Study Questionnaire. PDF (Adobe Acrobat) of the questionnaire used for data collection of cases.

**Additional file 2.** CONTROL GROUP Study Questionnaire. PDF (Adobe Acrobat) of the questionnaire used for data collection of controls.

## Data Availability

The data will not be publicly shared to protect the participants’ anonymity. The anonymized data used for analysis can be made available upon request to the corresponding author at the Institute of Tropical Medicine and International Health, Charité-Universitätsmedizin Berlin, Germany.

## Abbreviations

PrE/E: Preeclampsia and Eclampsia
PCMH: Princess Christian Maternity Hospital
UTIs: urinary tract infections
WHO: World Health Organization
DRC: Democratic Republic of Congo
MUAC: mid-upper arm circumflex
COR, AOR: crude and adjusted odds ratios
ANC: antenatal care
SLL: Sierra Leone Leones
