# Supplementary material for: Risk factors for preeclampsia and eclampsia at a main referral maternity hospital in Freetown, Sierra Leone: a case-control study
Source: BMC Pregnancy Childbirth. 2021 Jun 2;21:413. doi: 10.1186/s12884-021-03874-7 (PMC8173903; doi:10.1186/s12884-021-03874-7)
Supplement: Supplementary file 2 — Additional file 2. CONTROL GROUP Study Questionnaire. PDF (Adobe Acrobat) of the questionnaire used for data collection of controls. [file 12884_2021_3874_MOESM2_ESM.pdf]

Client Name (cut off at dotted line after data entry):____

....

CONTROL GROUP Study Questionnaire: Risk factors for Pre-Eclampsia/Eclampsia, PCMH

Hospital registration Number ____

Date of interview____

Study ID____

Staff name____

I. Sociodemographic/economic information

1. **Client age (years):** __________________
2. **Religion:** Christian □ Muslim □ Other □: ___________
3. **Ethnicity:** Mende □ Temne □ Limba □ Fullah □ Krio □ Other □: ___________
4. **Marital status**: Married □ Cohabit □ Single □ Widowed □ Divorced □
5. **Completed education:** None □ Primary □ Secondary □ Tertiary □
6. **Occupation:** Housewife □ Farmer □ Trader/Seller □ Student □ Other □:____________
7. **Income in SLL per month :** ______________ SLL
8. **Place of residence:** city □ suburb □ rural □
9. **Travel Time to Hospital(PCMH)**(min): __________**Travel cost to the hospital** (SLL) :________
10. **No. of children living at your home you take care of and age?**
    a. Own children (number and age)____
    b. Other children (number and age) ____
11. **Is there any pollution in your environment of daily living, e.g. working environment or home?**
    Yes (at least one mark below) □ No □ Don´t know □

Close to waste deposit □ Close to main road □ Cooking with open fire in closed room □
Working with chemicals (e.g. pesticides, paint, car components) □ Close to oil refinery □
Other sources of pollution □: ____

12. **Is the following present in your household:**

Electricity:	Yes □	No □	Pipe-borne water:	Yes □	No □	Mobile phone:	Yes □	No □
Radio:	Yes □	No □	TV:	Yes □	No □	Fridge:	Yes □	No □
Cattle:	Yes □	No □	Motor-bike, Car:	Yes □	No □	Mosquito- net:	Yes □	No □

13. **Main origin of drinking water:**

Bottled water □ Other packaged water □ Boiled pipe-borne water □ Unboiled pipe-borne water □
Boiled well water □ Unboiled well water □ Boiled bore whole water □ Unboiled bore whole water □
Other source □:________________________________

II. Recruitment information

1. **Confirm client was recruited postpartum**: □
2. **Date of delivery:** ____
3. **Confirm there was NO diagnosis of PE/E during this pregnancy:** □

Client Name (cut off at dotted line after data entry):____

....

III. Information in the case of client´s death

1. In case of reported death of client in study period, state date of death: ______________________

Specify, a. Cause of death: ____

b. Treatment: ____

c .Complications: ____

IV. Information of delivery

1. **Baby is:** live birth □ fresh stillbirth □ macerated stillbirth □ neonatal death □

   a. If stillbirth or neonatal death reason known?____________________________________
2. **Sex:** Female□ Male□
3. **Gestational week at delivery:** ____

   a. Preterm birth <37GW □
4. **Delivery mode:** Spontaneous vaginal □ Operation vaginal □ Planned CS □ Emergency CS □
5. **Apgar score(s)**: __________________
6. **Birth weight:** _____________________g
7. **Visible birth defects**? Yes □ No □ Don´t know □

   If yes, specify: ____
8. **Neonatal complications?** Yes □ No □ Don´t know □

   If yes, specify: ____

V. Pregnancy-related health information

1. **Current maternal weight:** ___________________kg, as of (date)______________________
2. **Visual evidence of overweight:** Yes □ No □ Don´t know □
3. **MUAC (mid upper arm circumference):** ________________cm
4. **No. of all your pregnancies (including this one):** _______________________________________
5. **Time interval to last pregnancy: months between dates of birth (or expected Date of Birth):**

   ____
6. **Have you ever experienced an abortion/miscarriage/stillbirth** Yes □ No □ Don´t know □

   a. If yes, how many? ____

   b. when (which year) ____

   c. If known, gestational week:____

   d. If known, state reason: ____
7. **Did you ever have an induced abortion?** Yes □ No □ **If yes,** which year(s)? _______________
8. **Current pregnancy a twin gestation?** Yes □ No □ Other (triplets) □___________________
9. **No. pregnancies with same partner of the current pregnancy:** Don´t know□

   First pregnancy with this partner□ Have one or more pregnancies with that partner already□
10. **Did you experience PE/E in a previous pregnancy?** Yes □ No □ Don´t know □

    a. If yes, which year(s) ____

    b. If yes, specify treatment____

    c. If yes, specify outcomes:____
11. **Known cases of PE/E in any other family member** (JUST Family related by blood) **?**

    Yes □ No □ Don´t know □

    a. If yes, specify who: Mother □ Aunt □ Sister □ Other □ ____________________________

    b. If yes, specify when: ____

Client Name (cut off at dotted line after data entry):____

....

12. **Did you experience one of the following during THIS pregnancy**?

Diarrhea (>2 weeks): Yes □ No □ Don't know □

Urinary tract infection: Yes □ No □ Don't know □

Anaemia: Yes □ No □ Don't know □

Gestational diabetes: Yes □ No □ Don't know □

Placenta praevia: Yes □ No □ Don't know □

STI/vaginal discharge : Yes □ No □ Don't know □

b. Specify any other medical condition or persistent complaint (what, when, treatment):

____

____

13. **Any Malaria episode during this pregnancy?** Yes □ No □ Don´t know □

If yes, reported Malaria Symptoms of client: asymptomatic □, or any of the following:

Fever □ Chills/Shivering □ Headache □ Vomiting □ Sweats □ Diarrhea □ Jaundice □

Other symptoms: ____

If yes, was diagnosis: symptom-based only □ lab-confirmed □ Don´t know □

If yes GW/Month of Pregnancy of Malariaepisode(s) : _______________GW(s)/Month(s)

If yes did you receive IPTp before this Malariaepisode(s) occured: Yes□ No□ Don´t know□

If yes was there any Treatment? Yes □ No □ Don´t know □

If yes, specify Treatment: ____

14. **Total number of ANC visits during this pregnancy**: ________

15. **Regular (at least 2 Doses) intake of IPTp (Fansidar)?** Yes □ No □ Don´t know □

If yes, specify how many Doses (1 Dose = 3 Pills) of IPTp (Fansidar) did you take?

1Dose □ 2Doses □ 3Doses□ >3 Doses □ specify how many :_______

If yes, specify when did you take IPTp (Fansidar) during pregnancy?

GW/Month of 1. Dose: ________ GW/Month

GW/Month of 2. Dose: ________ GW/Month

GW/Month of 3. Dose: ________ GW/Month

GW/Month of 4.Dose: ________ GW/Month

16. **Do you sleep under a Mosquito-net?** Yes □ No □ Don´t know □

If yes, how often did you sleep under a Mosquito-net <u>during this Pregnancy</u>?

never □ sometimes □ often □ daily □

Is the Mosquito-net treated with insecticide? Yes □ No □ Don´t know □

17. **Use of insect repellents indoor?** never□ sometimes □ often □ always □

a.Specify which kind of repellent: ____

18. **Did you experience ANY fever episode during this pregnancy?** Yes □ No □ Don´t know □

19. **Frequent (≥3 times a week) heavy physical work during pregnancy:** Yes □ No □

a. If yes: carry heavy weights □ farm work □ long distance walking (≥2 hours per day) □ prolonged standing(≥6 hours per day)□ manual laundry □ carry heavy weights on head □ other physical work:____

Client Name (cut off at dotted line after data entry):____

20. **Average sleep duration:** ≥6 hours per night □ 3-6 hours per night □ <3 hours per night □

a. **Sleeping quality during this pregnancy:**

daytime sleepiness □ snoring □ sleep-disordered breathing □ frequently waking up at night □

VI. **General health and behavior-related information**

1. **History of chronic Hypertension:** Yes □ No □ Don´t know □
   a. If yes since (year) ______________________________________
   b. If yes last measured blood pressure: _________ mmHg

2. **History of Diabetes** Yes □ No □ Don´t know □
   a. If yes since (year) __________________________
   b. If yes which Type? Type I □ Type II □ Don´t know □

3. **List all medical diagnoses you have or persistent complaints received during the PAST YEAR and treatments**: ____

   ____

   a. Visit of traditional healer? never □ sometimes □ often □
   b. Use of any traditional treatments? never □ sometimes □ often □
   If yes, please specify Herbs and Diagnosis you received during THIS Pregnancy:

   ____

   ____

4. **Family history of chronic Hypertension:** Yes □ No □ Don´t know □
   a. **If yes**, specify who (JUST Family related by blood): Mother□ Father□ Sibling□ Other□

5. **Family history of Diabetes:** Yes □ No □ Don´t know □
   a. **If yes,** specify who (JUST family related by blood): Mother□ Father□ Sibling□ Other□

6. **Do you smoke or used to smoke?** Yes □ No, never smoked □ Used to smoke, but quit □
   a. **If yes**, how many cigarettes per day /since when/ until when? ________________________

   ____

7. **Do you drink alcohol or used to drink alcohol?** Yes □ No, never □ Used to drink alcohol □
   a. If yes, how much per week/since when?____
   b. If yes, how much during this pregnancy?____

8. **Describe what you ate the last day at home BEFORE you came to the hospital (ALL MEALS!):**____

   ____

   ____

9. **Daily diet you eat every day includes calcium sources?**
   Yes, at least one (mark below) □ None □ Don´t know □
   Milk/dairy products □ Fish with bones □ Beans/Peas □ Groundnuts □
   Dark green leaves □ Calcium-supplemented food/juice □ Calcium Pills □ Other □

Client Name (cut off at dotted line after data entry):____

....

10. **General fruit intake:** never □ monthly □ every 2 weeks □ weekly □ daily □

11. **General vegetable intake:** never □ monthly □ every 2 weeks □ weekly □ daily □

12. **General meat intake:** never □ monthly □ every 2 weeks □ weekly □ daily □

13. **General animal products intake:** never □ monthly □ every 2 weeks □ weekly□ daily □
(e.g. cheese, milk, egg NO meat)

14. **Do you feel you have a lot of emotional stress in general?** Yes □ No □ Don´t know □
a. If yes, specify reasons: money problems□ personal conflicts□ domestic violence□
family conflicts □ death □ Other reasons□:___________________________________
____
Describe common stressful events: ____
Frequency of such stressful events: <3 times a week □ ≥3times a week □ daily □

Una Tenky 😊
